# Epigallocatechin Gallate: A Review of Its Beneficial Properties to Prevent Metabolic Syndrome

**DOI:** 10.3390/nu7075230

**Published:** 2015-07-07

**Authors:** Samuel Legeay, Marion Rodier, Laetitia Fillon, Sébastien Faure, Nicolas Clere

**Affiliations:** 1 INSERM U1063, Stress oxydant et pathologies métaboliques, LUNAM Université, Angers 49045, France; E-Mails: samuel.legeay@etud.univ-angers.fr (S.L.); marion.rodier@univ-angers.fr (M.R.); sebastien.faure@univ-angers.fr (S.F.); 2 UFR des Sciences Pharmaceutiques et Ingénierie de la Santé, Département pharmacie, Laboratoire de pharmacologie, LUNAM Université, Angers 49045, France; E-Mail: laetitia.fillon@gmail.com

**Keywords:** metabolic syndrome, green tea, epigallocatechin gallate, EGCG, endothelial dysfunction, cardiovascular diseases

## Abstract

Obesity and being overweight are linked with a cluster of metabolic and vascular disorders that have been termed the metabolic syndrome. This syndrome promotes the incidence of cardiovascular diseases that are an important public health problem because they represent a major cause of death worldwide. Whereas there is not a universally-accepted set of diagnostic criteria, most expert groups agree that this syndrome is defined by an endothelial dysfunction, an impaired insulin sensitivity and hyperglycemia, dyslipidemia, abdominal obesity and hypertension. Epidemiological studies suggest that the beneficial cardiovascular health effects of diets rich in green tea are, in part, mediated by their flavonoid content, with particular benefits provided by members of this family such as epigallocatechin gallate (EGCG). Although their bioavailability is discussed, various studies suggest that EGCG modulates cellular and molecular mechanisms of various symptoms leading to metabolic syndrome. Therefore, according to *in vitro* and *in vivo* model data, this review attempts to increase our understanding about the beneficial properties of EGCG to prevent metabolic syndrome.

## 1. Introduction

Metabolic syndrome (MS) is a major and growing public-health and clinical challenge worldwide whose affects approximately 25% of the adult in the world [1,2]. MS increases the risks of developing type 2 diabetes (5-fold), stroke (2- to 4-fold), myocardial infarction (3- to 4-fold) and the risk of death (2-fold) regardless of a previous history of cardiovascular events [2,3]. MS is defined by a multitude of pathophysiological disorders comprising abdominal obesity, insulin resistance, high blood pressure, and dyslipidemia. Several scientific organizations have attempted to formulate working definition of the syndrome [2]. Although each definition possesses common features, the major problem with these definitions is their applicability to the different ethnic groups, especially to define obesity cut-offs. This is particularly evident for the risk of type 2 diabetes which is apparent at much lower levels of obesity in Asians compared to Europeans [2]. In this context, the International Diabetes Federation (IDF) proposed a new set of criteria with ethnic specific cut-offs. However, for many years, the most commonly accepted definition is that of the National Cholesterol Education Program Adult Treatment Panel (NCEP ATP III). Thereby, the diagnosis of MS is established when the patient describes at least three of the following criteria: abdominal obesity, hyperglycemia, elevated blood pressure and dyslipidemia. Abdominal obesity is defined by a waist circumference cut-off greater than 102 cm for men and 88 cm for women, and hyperglycemia is defined by a fasting plasma glucose greater than 5.6 mmol/L (100 mg/dL) and/or the existence of a symptomatic treatment (such as metformin or insulin in the most advanced forms). Furthermore, hypertension is diagnosed when patients present a systolic and/or diastolic blood pressure greater than 130 mmHg and 85 mmHg, respectively, and/or specific treatment (angiotensin-converting enzyme (ACE) inhibitors, calcium channel blockers). Finally, dyslipidemia is established when plasma triglycerides (TG) are greater than 1.7 mmol/L (150 mg/dL), and/or high-density lipoprotein cholesterol (HDL-C) are lower than 1.0 mmol/L (40 mg/dL) for men and 1.3 mmol/L (50 mg/dL) for women, and/or when patient is already receiving symptomatic treatment (fenofibrate).

Dietary, pharmacological and surgical strategies have been developed in the last decade to prevent metabolic syndrome. Recently, beneficial effects of a polyphenol-enriched diet have been reported in the prevention of this metabolic disease [4]. Polyphenols represent an important group of phytochemicals found in plants and more than 8000 polyphenolic compounds are currently known [5]. According to the number of phenolic rings, polyphenols are classified into four categories: phenolic acids, flavonoids, stilbenes and lignans. Flavonoids represent 60% of dietary polyphenols and they are classified into seven groups: flavones, flavonols, flavanones, isoflavones, flavanols, anthocyanins and chalcones.

Evidence from epidemiologic studies supports a potential role for some flavonoids in the reduction of cardiovascular risk. For instance, flavonoids are able to prevent against endothelial dysfunction through averting oxidation of low-density lipoproteins (LDL) [6], platelet aggregation and adhesion [7], and smooth muscle cell migration and proliferation [8]. Moreover, according to recent data aiming to evaluate association between dietary flavonoid intake and cardiovascular risk through analyses of prospective cohort studies, it has been reported that intakes of epigallocathechin gallate (EGCG) (relative risk: 0.87; 95% confidence interval: 0.80, 0.95) were inversely associated with the risk of cardiovascular diseases [9]. Based on these considerations, this review attempts to (i) describe green tea polyphenols, their main pharmacokinetic properties and theirs structure/activity relationship explaining its antioxidant effects and (ii) to explain the beneficial properties of EGCG to prevent pathological disorders defining MS such as obesity, insulin resistance, dyslipidemia and hypertension.

## 2. Green Tea Polyphenols

Green tea, derived from the tea plant *Camellia sinensis* is considered as the most consumed beverage in the world [10]. Originally found in China, the tea plant is now cultivated in over 30 countries and it is estimated that about 120 mL per person of tea beverage is consumed every day [11]. According to data obtained by high performance liquid chromatography (HPLC), green tea leaves are composed of 26% fibres, 15% proteins, 2%–7% lipids, 5% vitamins and minerals, secondary metabolites as 1%–2% pigments, 30%–40% polyphenols of which at least 80% flavonoids and 3%–4% methylxanthines [10,12,13]. This composition can vary depending on growing conditions like, geographical location (climate, soil, *etc.*), agricultural practices (fertilizers, deadheading, *etc.*) and the properties of the plant itself (variety, age of the leaf, position of the leaf on the harvested shoot, *etc.*) [10,13].

Tea infusion is as a hot aqueous extraction containing more hydrosoluble compounds than liposoluble derivatives. An increase of time and temperature would theoretically enrich beverage in green tea leaf components. However, it has been reported that the optimum extraction occurs for water at 80 °C and for 5 min to 15 min for green tea leaves in powder or in bag form, respectively. Indeed, degradation of bioactive compounds is suggested beyond these times and temperatures [14].

**Figure 1:**
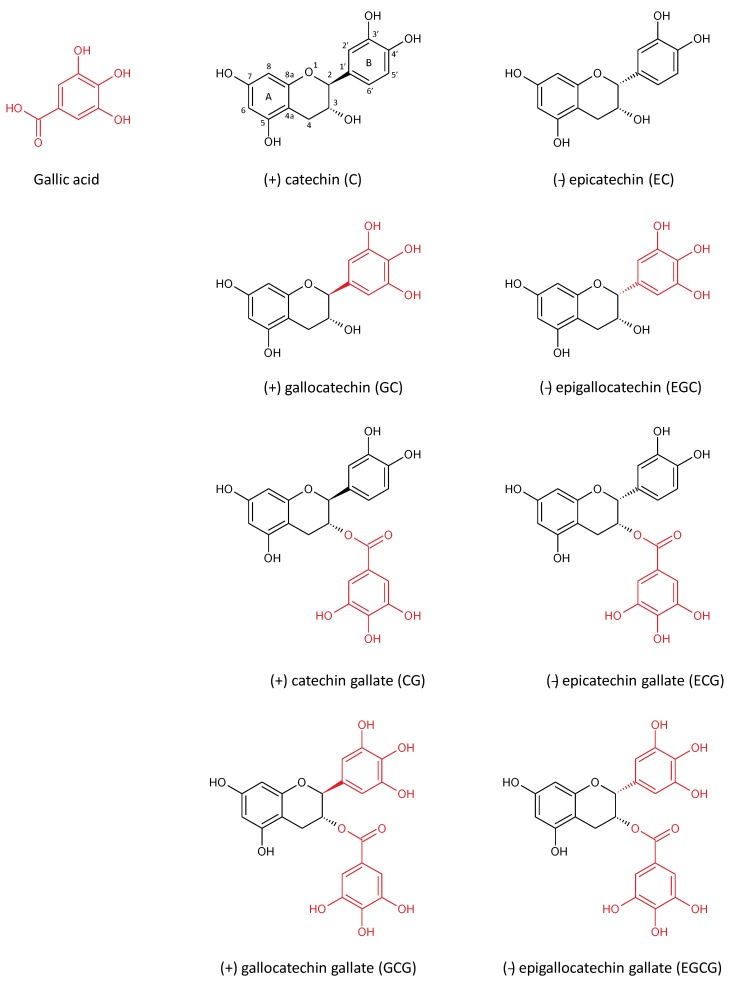
Molecular structure of gallic acid and catechins.

During the past decade, the health-promoting effects of green tea and its polyphenols have been intensively investigated. Flavonoids are the most important polyphenols in tea leaves. They represent the major component of green tea infusions, with a percentage between 37% and 56% of weight of solid extracts [10]. Furthermore, green tea beverages also contain carbohydrates, amino acids, organic acids, methylxanthines, minerals, polymers and tannins and traces of volatiles compounds (Table 1) (for review see [13]). Catechins are the main flavonoids found in green tea beverage [15]. They are constituted by a 2-phenylchromane skeleton substituted in 3, 5, 7, 3′ and 4′ positions with hydroxyl groups. During the biosynthesis, if the B-ring derives from the gallic acid synthon, the catechin is also substituted in 5′ position with a hydroxyl group and thus named “*gallo*” catechin. Moreover, the hydroxyl group in 3′ position can be esterified with the gallic acid, thus forming catechin “*gallate*”. Finally, the levorotatory (2r, 3r) compounds are considered as “*epi*” catechins while the dextrorotatory (2s, 3r) compounds are simply named “*catechins*”. Thus, with these combinations, eight molecular structures can be distinguished (Figure 1).

Among catechins, only EGCG has an interest in the field of medicinal chemistry. Indeed, EGCG is the most abundant catechin in green tea infusions (for review see [13,15]) and it is considered as one of the most active molecules known for their antioxidant properties [16] (Table 2).

**Table 1:** Mean composition (% weight of solid extract) of green tea infusion determined by high performance liquid chromatography (HPLC).

Compound	% Weight of Solid Extracts
Flavonoids	37–56
Carbohydrates	10–15
Amino acids	8–12
Organic acids	7.5–9.5
Methylxanthines	7–9
Minerals	6–8
Polymers and tannins	3–4
Volatiles	Traces

**Table 2:** The composition of polyphenols in green tea leaves determined by high performance liquid chromatography (HPLC) (adapted from [14]).

Catchins	ConntraIion (mg/mL, Mean ± SD)
(+) catechin (C)	19.70 ± 0.10
(−) epicatechin (EC)	123.43 ± 0.13
(+) gallocatechin (GC)	51.10 ± 1.13
(−) epigallocatechin (EGC)	279.87 ± 1.87
(+) catechin gallate (CG)	nd
(−) epicatechin gallate (ECG)	108.55 ± 0.11
(+) gallocatechin gallate (GCG)	3.90 ± 0.06
(−) epigallocatechin gallate (EGCG)	324.54 ± 0.17
TOTAL	911.09

## 3. Properties of EGCG in the Control of Oxidative Stress

In several *in vitro* studies, EGCG has been found to have the highest antioxidant activity compared to others catechins [17]. Indeed, EGCG has shown an efficient ability in scavenging free radicals species, notably through achievement of the ATBS^•+^ radical scavenging test [17]. One hypothesis to explain these properties is a low reduction potential of EGCG due to its high capacity for giving an electron [16]. Electron delocalization in the molecular structure is described as a property of polyphenolic compounds which could in part be responsible for their antioxidant activity [18]. In the catechin skeleton, the saturation of the heterocyclic ring prevents electron delocalization between the A and the B ring. Thus, for green tea catechins, the antioxidant potential mainly comes from the strong presence of hydroxyl groups in their molecular structures. EGCG, with 8 hydroxyl groups notably in 3′, 4′ and 5′ positions and with a gallate moiety in C-3 is a better electron donor than the others catechins and thus the best scavenger of free radicals species [16,17].

**Figure 2:**
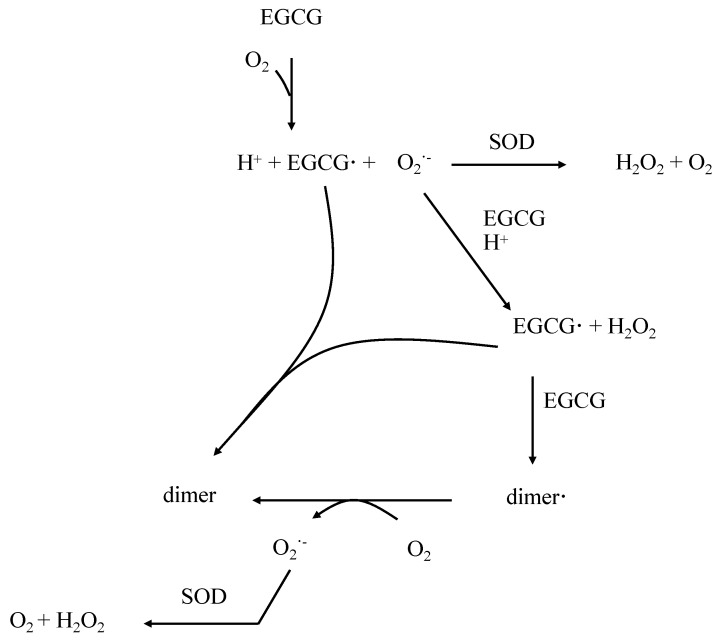
Mechanism of epigallocathechin gallate (EGCG) auto-oxidation and dimerization adapted from [30].

Moreover, the antioxidant activity of EGCG is also due to its ability to chelate metal ions. Troubles in metals homeostasis can lead to an oxidative stress which appears in chronic diseases like diabetes, cardiovascular disease and atherosclerosis [19]. It has been reported that EGCG can chelate metals like iron (Fe) [20], copper (Cu) [21,22], chromium (Cr) [23] and cadmium (Cd) [24,25]. The phenolic groups notably at the B ring are mainly suspected to be responsible for this property [18]. The chelation of metal ions by EGCG is however considered as a minor mechanism in the antioxidant action compared to its free radical scavenging capacity [25,26]. Interestingly, it has been noted that EGCG, in addition to chelate ions, also reduces Fe (III) and Cu (II) in Fe (II) and Cu (I), respectively [27,28]. Fe (II) and Cu (I) are involved in Fenton reaction, with production of radical oxygen species (ROS) [19]. Furthermore, as it is commonly found with antioxidant polyphenols, EGCG may generate ROS *in vitro*, probably via auto-oxidation and dimerization [29,30,31]. Indeed, Hou Z. *et al.* have proposed a mechanism of EGCG auto-oxidation through a classical pathway including transfer of electron [30]. Therefore, it has been proposed that EGCG is oxidized in EGCG radical (EGCG·) through a sharing of an electron with the oxygen O_2_ thus producing superoxide anion (O_2_^−^). Then, this EGCG radical can form a homo-dimer with another EGCG radical or a dimer radical (dimer·) with another EGCG. Finally, the neutralization of the dimer radical can occur via production of superoxide anion from the O_2_. Thus, the conversion of O_2_^−^ in H_2_O_2_ by the superoxide dismutase (SOD) makes this enzyme indispensable for the inhibition of the propagation of the chain reactions [30] (Figure 2). The 3’,4’,5’-trihydroxy function and the aromatic B ring mainly supports this ability for EGCG to share an electron. Furthermore, while the interaction between SOD activity and EGCG is not clearly established, it has been reported on rats with acetic acid-induced colitis an increases activity of SOD in EGCG-treated rats in comparison with placebo or control rats. To explain these data, authors suggested that the enhanced antioxidant activity of EGCG might be related to its special molecular structure appeared to be important for these actions, which includes two catechol groups, three gallate groups, and two hydroxyl groups [32]. This structure would explain the increase in gene expression induced by EGCG since several studies have reported an increase of *sod* gene expressions induced by this catechin [33,34].

As previously described, EGCG is a natural antioxidant and most of its pharmacological properties are considered to be due to their antioxidant effects. These properties are beneficial to prevent various diseases associated with an increased oxidative stress. However, it has been reported that EGCG has pro-oxidant properties, mainly in cancer cells where it contributes to induce apoptosis [35]. One of the first studies showing the pro-oxidant properties of EGCG has found that 4 mM EGCG is able to favor hydroxyl radical and superoxide anion productions to promote tumor cells apoptosis. Further, it has been reported in this study that copper mediated oxidation of EGCG possibly leads to the formation of polymerized polyphenols. It was indicated that copper oxidized catechins were more efficient prooxidants as compared with their unoxidized forms [28]. Furthermore, toxic effects of EGCG observed *in vivo* following consumption of dietary supplements in humans [36] and administration of tea extracts in animal studies were considered as based on its pro-oxidant activities of EGCG [37]. A study conducted on NCr *nu/nu* mice, xenografted with human lung cancer cells, has demonstrated that intraperitoneal treatment with 30 mg/kg EGCG increases significantly ROS production [38].

## 4. Pharmacokinetical Properties of EGCG in Humans

Pharmacokinetic parameters of green tea polyphenols, particularly EGCG, have been well investigated in rodents but some of these remain unclear in humans [39]. Few pharmacokinetic studies have evaluated the bioavailability of EGCG. However, it has been revealed a very low absorption of EGCG (probably <5%) and an average T_max_ of 2 h after *per os* administration [40,41,42]. Green tea catechins are predominantly absorbed intestinally, in the jejunum and the ileum, via a paracellular diffusion through epithelial cells [43]. Once absorbed, EGCG is found in plasma in large proportion (>75%) in a free form [39,44]. The only calculated apparent distribution volume 0.15 L/kg in rat theoretically reveals a weak distribution of EGCG [45]. Despite this low distribution, EGCG seems to diffuse well through tissues in the body. Indeed, EGCG has been found in fetuses and placenta of pregnant rats [46] and in the brain through crossing the blood-brain-barrier [47,48]. EGCG is metabolized on one hand through methylation by the catechol-*O*-methyltransferase (COMT) producing predominantly the primary metabolite di-methoxyl-EGCG (di-OMe-EGCG) [49]. On the other hand, EGCG can be glucurono- and/or sulfo-conjugated [40]. In addition, it is now well established that EGCG can also be metabolized by the intestinal microbiota [50,51]. The half-life is around 3 h, according to the association with others catechins, in a purified form or from tea infusion [40,42]. EGCG metabolites are both excreted through biliary and urinary pathways. However, only traces of EGCG are detected in urine after oral administration [39,45,49,52]. Furthermore, EGCG can be reabsorbed from the intestine through enterohepatic re-circulation.

Although the metabolic transformation of catechins in humans is well understood, relatively little is known about the biological effects of catechin metabolites. However, several studies seem to agree on possible antioxidant properties of both EGCG and its metabolites. Thus, it has been found that O-methylated derivatives of (−)-epicatechin are able to inhibit the peroxynitrite-mediated nitrotyrosine formation [53]. Furthermore, in human skin fibroblasts, it has been shown that 3′-O-methyl-epicatechin prevents UVA-induced oxidative damage through an enhancement of HO-1 activity [54]. Interestingly, it has been found that HUVEC have the capacity to convert (−)-epicatechin into methyl derivatives, which inhibited NADPH oxidase activity [55].

It has been emphasized that the low bioavailability of EGCG should be considered for the extrapolation of *in vitro* studies to *in vivo* situations. This point is currently debated, notably because *in vitro* studies are often performed with non-physiological concentrations of EGCG [56]. However, numerous factors have been identified to enhance or to diminish its bioavailability [57]. However, the benefits of green tea consumption in humans result from long-term exposition whereas *in vitro* studies supply short-term effects [58]. Thus, *in vitro* data could not necessarily be related with important relevance to clinical data, so it can suggest that *in vitro* studies often converge with epidemiological studies [56,59].

## 5. Roles of EGCG in Obesity

Obesity is principally the consequence of a positive energy balance driven by increased calorie-dense food consumption and reduced physical activity. Adipose tissue is composed of adipocytes, pre-adipocytes, immune cells and endothelial cells. It can respond rapidly and dynamically to alterations in nutrient excess through adipocytes hypertrophy and hyperplasia [60]. Adipose tissue has long been considered as an organ of lipids storage and mobilization. It has recently been identified as an endocrine organ because of its ability to secrete a large amount of biologically active metabolites as glycerol, free fatty acids (FFA), and pro-inflammatory mediators such as tumor necrosis factor alpha (TNFα), interleukin-6 (IL-6) or leptin [61,62,63].

FFA are involved in the increase in glucose, triglycerides and VLDL synthesis in adipocytes. In addition, reducing insulin sensitivity of skeletal muscle, FFA inhibit glucose uptake and consequently raise circulating glucose levels which increase the pancreatic insulin secretion and lead to hyperinsulinemia [64]. Secretion of TNFα and IL-6 by adipocyte and macrophage are increased and promotes insulin resistance and lipolysis in adipose tissue [65]. Leptin is a regulator of food intake, body weight and fat mass. The plasma levels of leptin are positively correlated with the degree of adiposity in healthy and obese individuals [66,67,68]. Besides, leptin is known to be a NO-dependent vasodilator and an endothelium-independent vasoactive agent [69,70]. Thereby, acute hyperleptinemia induces vasorelaxation that seems to contradict the hypertension observed during obesity. This could be explained by a study showing that leptin receptors in coronary arterioles are downregulated in high-fat fed sedentary mice, leading to endothelial dysfunction [71]. In addition, leptin may impair endothelial function through oxidative stress by increasing the formation of ROS that reduce the bioavailability of NO and upregulate proinflammatory cascades including adhesion and chemotactic pathways in endothelial cells [72,73,74]. Various studies have described the beneficial properties of EGCG to prevent obesity. Thus, Snoussi *et al.* reported that oral administration of EGCG decoction daily to male Zucker rats fed a high fat diet (22% fat, 43% carbohydrates and 21% proteins) resulted in reduction of body weight within 1 week. In addition, rats treated with EGCG had significantly lowered blood lipids (50% triglycerides and 25% cholesterol) and blood glucose (15%) concentrations. Furthermore, it has been shown that EGCG is able to control glucose homeostasis through a reduction of intestinal SGLT-1/GLUT2 ratio and an enhancement of adipose GLUT4 [75].

Fiorini *et al.* have studied the effects of EGCG on obesity and hepatic steatosis in leptin-deficient *ob/ob* mice. Treatment with 85 mg/kg EGCG for 5 days resulted in decreased body weight gain compared to control mice. EGCG treatment also reduced significantly total hepatic fat content (22.7% ± 11.0%), increased hepatic energy stores and hepatic antioxidant activity through an enhancement of glutathione level in EGCG-treated mice, compared to control mice. Lipophilic oil red O stain showed that EGCG treatment decreased hepatic steatosis through a significant decrease of palmitic and linoleic acids [76].

Various mechanisms have been proposed to explain the anti-obesity properties of EGCG. Several groups have reported a modulation of dietary lipid absorption by EGCG treatment. In a study conducted on male C57BL/6 mice fed with high fat diet supplemented with EGCG, the anti-obesity properties of this flavonoid was explained by a decreased of food digestibility affecting substrate metabolism of intestinal mucosal and liver, leading to increased post-prandial fat oxidation and reduced incorporation of dietary lipids into tissues [77]. Furthermore, EGCG has been reported to inhibit pancreatic lipase. Thus, in obese C57BL/6 mice fed with high fat diet, it has been found that treatment with 0.32% EGCG for 6 weeks favored a significant decrease in body weight (44%) in comparison with control mice. To explain these beneficial effects, it has been suggested that EGCG is able to inhibit pancreatic lipase [78]. Recently, the molecular interactions between EGCG analogs and pancreatic lipase have been described by Wang *et al.* These authors confirmed that EGCG had different effect on activity, conformation, thermodynamics and kinetics of pancreatic lipase suggesting that EGCG could contribute to the development of natural effective pancreatic lipase inhibitors to prevent human obesity [79]. Otherwise, it has been demonstrated that a high consumption of EGCG inhibited pancreatic lipase *in vitro* and suppressed postprandial serum triglycerides in a dose-dependent manner [80]. To explain the mechanism of action, it has been proposed that the hydroxyl moieties of EGCG interact with the hydrophilic head group of phosphatidylcholine at the exterior of a lipid emulsion by forming hydrogen bonds. These interactions may lead to formation of cross-links followed by covalescence of the emulsion droplets [81]. Several studies have examined the effects of EGCG on fat metabolism and particularly in β-oxidation. Recently, it has been suggested that EGCG could alleviate fat deposition in broilers through inhibiting fat anabolism and stimulating lipid catabolism in broilers. Then, the supplementation of old male Ross 308 broiler chickens by EGCG for 4 weeks showed a significant downregulation of the expression of fatty acid synthesis and an upregulation of genes involved in fatty acid β-oxidation and lipolysis. Simultaneously, the activities of fatty acid synthase and acetyl CoA carboxylase were significantly decreased whereas the activity of carnitine palmitoyl transferase-1 was notably elevated by EGCG [82]. To understand the influence of EGCG on fatty acid metabolism, a first study performed on high fat diet mice revealed that EGCG modulates body weight gain through an increase of nuclear respiratory factor (*nrf*) 1, medium chain acyl CoA decarboxylase (*mcad*), uncoupling protein (*ucp*) 3, and peroxisome proliferator responsive element (*ppar*)-α genes [83]. Furthermore, another *in vitro* study, conducted on human hepatoma HepG2 cells, demonstrated that EGCG inhibited the HMG-CoA lyase activity reducing acetoacetate production and then, prevents ketoacidosis [84]. These studies suggest that EGCG is able to prevent obesity through a modulation involving different organs such as adipose tissue or liver, for example.

## 6. Involvement of EGCG in Insulin Resistance

Insulin resistance is the key pathophysiological feature of the MS, an important risk factor for cardiovascular disease and diabetes [85]. This pathophysiological condition is defined by a normal insulin concentration that does not adequately produce a normal insulin response in the peripheral target tissues such as adipose, muscle, and liver. The inability of the organism to overcome this insulin resistance leads to hyperinsulinemia, hyperglycemia and type 2 diabetes [86]. If hyperinsulinemia does not allow the maintenance of normoglycemia, it may cause an overexpression of insulin activity in some normally sensitive tissues. In these conditions, the effects of insulin are mediated by an endothelial dysfunction explained in part, by the increased production of endothelin-1 (ET-1) which promotes vasoconstriction, oxidative stress, cell-growth and mitogenesis, and by the activation of the vascular tissue renin–angiotensin system (RAS) [87,88].

Because EGCG has been suggested as a therapeutic agent for the treatment of diabetes, several studies have evaluated the role of this flavonoid in the control of blood glucose concentration. In a study performed on young *db/db* mice fed with diet enriched with EGCG, it has been reported that EGCG improves glucose tolerance and increases glucose-stimulated insulin secretion by preserving islet structure in comparison with control mice [89]. One hypothesis to explain these beneficial effects would be a potentiation of anti-inflammatory properties induced by this flavonoid. In female non-obese diabetic mice treated with 0.05% EGCG in drinking water, a delay of the onset of type 1 diabetes explained by a significant increase of anti-inflammatory cytokine IL-10 has been reported [90]. This hypothesis has been confirmed in another *in vitro* study conducted on RINm5F cells exposed to a combination of recombinant interleukin-1beta (IL-1β), TNF-α, and interferon gamma (IFN-γ), with or without EGCG pretreatment for 24 h. EGCG pretreatment prevented the inflammation-induced destruction of β-cells through a decrease of both mitochondrial reactive-oxygen species production and mitochondrial membrane potential and cytochrome *c* release [91].

In addition to its effects on hyperglycemia, EGCG has also been examined for its effects on diabetes-related comorbidities. Thus, the beneficial effects of this flavonoid have been evaluated in diabetic retinas from Wistar rats and in retinal Müller cells under diabetic conditions. This study revealed that EGCG was able to protect retina against glucose toxicity through an antioxidant mechanism [92]. Furthermore, diabetic nephropathy is one of the most serious complications in diabetes mellitus. Glucose-dependent pathways are activated within the diabetic kidney, such as increasing oxidative stress, polyol formation, and advanced glycation end-products (AGE) accumulation. In a model study of rats in which diabetes has been induced by subtotal nephrectomy and streptozotocin injection, it has been shown that oral administration of EGCG for 50 days suppressed hyperglycemia, proteinuria and lipid peroxidation. Otherwise, it reduced renal advanced glycation end-product accumulation and its related protein expression in the kidney cortex as well as associated pathological conditions [93]. Some recent studies have investigated the properties of EGCG in diabetic neuropathy, the most common complication of diabetes induced by an enhancement of oxidative stress. On streptozotocin-induced diabetic rats, it has been reported that treatment with EGCG for 10 weeks normalized the increase of 8-hydroxy-2′-deoxyguanosine, a marker of oxidative stress, and neuronal hypersensitivity. These findings suggest original properties of EGCG in the prevention of diabetic neuropathy [94].

Among the various mediators involved in the complications of diabetes, osteopontin plays a key role. Osteopontin, a profibrotic adhesion molecule, has been expressed in the renal tubules and glomerular epithelial cells [95]. Although osteopontin is reported to facilitate recovery from acute tubular injury, it has been shown in renal damage associated with inflammatory glomerulonephritis, obstructive uropathy and tubulointerstitial disease [96]. Based on these findings, osteopontin may be considered as a prognostic marker of diabetic nephropathy. Therefore, a recent study conducted on streptozotocin-induced diabetic nephropathy in mice showed that EGCG 100 mg/kg might provide an effective protection against diabetic nephropathy by osteopontin suppression suggesting that this flavonoid may provide supportive aid for management of *diabetes mellitus* patients with nephropathy [97].

Although the effects of EGCG on type 1 diabetes are interesting, the recent increases in the incidence of obesity make understanding the effects of EGCG against type 2 diabetes very important. As such, in studies conducted on non-obese type 2 diabetic Goto-Kakizaki rats, it has been found that EGCG treatment improved glucose tolerance and glucose homeostasis in GK rats, and reduced oxidative stress and mitochondrial dysfunction in skeletal muscle. These ameliorations have been explained through a down-regulation of the ROS-ERK/JNK-p53 pathway, a reduction of oxidative stress and inhibition of mitochondrial loss and dysfunction [98]. Recently, the direct effects and mechanisms of EGCG on glucose and lipid metabolism have been elucidated in HepG2 cells. Interestingly, it has been reported that EGCG enhanced glycogen synthesis in a dose-dependent manner and inhibited lipogenesis through an enhancement of phosphorylated AMP-activated protein kinase α and acetyl-CoA carboxylase expressions [99]. Otherwise, it has been suggested that EGCG improved insulin sensitivity of HepG2 treated with high glucose, preventing or delaying a potential hepatic dysfunction through the attenuation of the insulin signaling blockade and the modulation of glucose uptake and production. These last findings have been explained by (i) a decrease of tyrosine-phosphorylated and total levels of insulin receptor, insulin receptor substrate (IRS)-1 and -2 triggered by high glucose and (ii) a prevention of the inactivation of the PI3K/AKT pathway and AMPK, as well as a diminution of GLUT-2 levels induced by high glucose [100].

A growing body of evidence indicates that toll-like receptor 4 (TLR4) is a cell surface receptor, a natural immune and pattern recognition receptor expressed in most tissues of the body, that plays a central role in the occurrence of chronic inflammatory diseases, such as obesity-related insulin resistance [101,102]. In a recent study conducted on high-fat diet rats, it has been reported that EGCG significantly decreased free fatty acids, fasting insulin, homeostasis model assessment-insulin resistance index, and epididymal fat coefficient, and increased glucose infusion rate compared to control rats. Furthermore, this study revealed that EGCG attenuated inflammation by decreasing the content of macrophages, interfered the toll-like receptor 4 mediated inflammatory response pathway, thus, improved insulin signaling in adipose tissues [103].

## 7. Influence of EGCG in Dyslipidemia

Dyslipidemia is characterized by lipids disturbance including an elevation of lipoproteins containing apolipoprotein B (apoB), elevated TGs, increased levels of small particles of LDL, and low levels of HDL- cholesterol. Dyslipidemia, associated with MS, consists of a reduction of HDL-cholesterol and an increase in plasma LDL and TG [104]. As previously described, obesity and insulin resistance play a key role in the development of dyslipidemia associated with MS. Indeed, an elevated lipolysis is observed in the adipose tissue of obese patients, resulting in an important release of FFA and consequently in an increase in TG synthesis and very low density lipoprotein (VLDL) production. Insulin resistance takes part in this process by decreasing ApoB degradation [105,106] and lipoprotein lipase concentration in peripheral tissue that contributes to hypertriglyceridemia and VLDL overproduction [107]. Hypertriglyceridemia, and indirectly insulin resistance, is related to changes in HDL composition and metabolism, leading to an increased clearance of HDL from the circulation. In addition to HDL, the composition of LDL is also modified and patients show a predominance of small dense LDL [108] potentiating the atherogenic risk associated to MS.

Several studies have investigated the relationship between EGCG and the level of blood lipoprotein. Many of them concluded that EGCG is able to reduce total blood cholesterol, LDL-cholesterol and triglycerides. Thus, from a DNA microarray analysis performed on HepG2 hepatocytes treated with 10 μM or 25 μM EGCG, it has been reported an up-regulation of *ldlr* mRNA and a significant decrease of extracellular apoB levels suggesting beneficial properties of EGCG to improve cholesterol metabolism [109]. Recently, to confirm these first data, the metabolic profile response to administration of EGCG has been studied in high-fat-fed mice. Then, it has been noted that treatment with 50 mg/kg EGCG for 60 days is able to decrease adipose tissue, triglycerides and HDL-cholesterol only in high-fat diet mice [110]. The preventive role of EGCG from hypercholesterolemia has been described in a recent study [111] conducted on Sprague Dawley rats treated with 550 mg/500 mL EGCG. Furthermore, cholesterol and LDL have been reduced by drink containing EGCG in comparison with control drinks. *In vitro* mechanistic studies on EGCG and prevention of dyslipidemia have focused on the antioxidant properties of this polyphenol. Then, it has been reported that EGCG can prevent oxidation of LDL cholesterol *in vitro* [112]. For instance, 1 to 10 g/mL EGCG was shown to dose-dependently reduce LDL oxidation induced by Cu^2+^ [113].

## 8. Roles of EGCG in Hypertension

Obesity and insulin resistance are now recognized to be associated with hypertension [114,115]. As previously described, these pathophysiological situations are favored by an endothelial dysfunction characterized by an enhancement of RAS mediators expression [116,117,118] and a decrease in NO bioavailability.

Endothelial dysfunction is characterized by an impaired endothelium-dependent vasodilation inducing a reduced arterial compliance and an increase of inflammation and pro-thrombotic properties [119,120].

The pathophysiology of endothelial dysfunction is complex and involves multiple mechanisms. First of all, reduction of NO availability dependent of oxidative stress is frequently described. Then, NO reacts with O_2_^−^ to form peroxynitrite (ONOO^−^) [121] a cytotoxic oxidant which alters protein function, oxidizes LDL and leads to a reduced activity of endothelial nitric oxide synthase (eNOS). Besides, ROS upregulated adhesion molecules (ICAM and VCAM) and chemotactic molecules (MCP-1), resulting in establishment of pro-inflammatory state in the vessel wall.

Oxidative stress is intimately linked to inflammation because it may amplify vascular inflammation signaling pathways [122,123,124].

Obesity, diabetes/insulin resistance, hypertension and MS are known to induce endothelial dysfunction [125,126,127,128] which is an important early event in the pathogenesis of atherosclerosis [127] and is consequently a starting point of cardiovascular diseases associated with MS [3].

Thus, endothelial dysfunction is one of the characteristics of hypertension and hypertension is a hallmark of endothelial dysfunction. Therefore, many studies have evaluated the beneficial properties of EGCG to improve endothelial function. One of endothelial dysfunction models is based on the lipid peroxidation induced by asymmetric dimethylarginine (ADMA) [129]. ADMA is synthesized by the protein arginine methyltransferase (PRMT) using *S*-adenosylmethionine as methyl group donor. Conversely, it is degraded by dimethylarginine dimethylamino hydrolase (DDAH), an oxidant-sensitive enzyme with sulfhydryl groups in its structure [130]. ADMA and DDAH are widely distributed in endothelial cells [131] and ADMA is thought to induce endothelial dysfunction through an inhibition of eNOS by competing with L-arginine [132]. Thus, in HUVEC treated with 100 μg/mL oxidized low density lipoprotein (ox-LDL), EGCG (10 and 100 mg/mL) significantly increased the level of nitrite/nitrate and the activity of DDAH suggesting that EGCG improved endothelial dysfunction by decreasing level of ADMA and by enhancing endothelial nitric oxide production. Moreover, in the same study, in a model of endothelial dysfunction induced by LDL in rats, it has been confirmed that EGCG (10 or 50 mg/kg) significantly attenuated the inhibition of vasodilator response to acetylcholine through a decreased serum nitrite/nitrate level associated with a decrease of the elevated levels of ADMA [133].

Furthermore, to understand the structure-activity relationship causing increased production of NO, a recent study has examined the effect of selective replacement of hydroxyl functions on either the B or D ring on the EGCG-induced phosphorylation of AKT and eNOS, formation of ROS and NO in cultured coronary artery endothelial cells, and endothelium-dependent relaxation of coronary artery rings. Interestingly, it has been found that the hydroxyl group at the 3′ position of the gallate ring is essential and, also, to some extent, the two hydroxyl groups at positions 3′ and 4′, for the PI3-kinase/AKT-dependent phosphorylation of endothelial NO synthase leading to the subsequent NO-mediated vascular relaxation [134].

Other pathophysiological mechanisms may explain hypertension. Thus, the kidneys increase sodium reabsorption, the heart increases cardiac output, and arteries respond with vasoconstriction resulting in hypertension. Secondly, compression exerted by the visceral fat on the renal parenchyma may cause hemodynamic disturbances [135]. Finally, adipocytes are able to produce aldosterone in response to angiotensin II and may be considered as a miniature renin-angiotensin-aldosterone system [136]. All this mechanisms may contribute to the development of hypertension in patients with insulin resistance and/or obesity.

As previously shown, the RAS plays a major role in regulating blood pressure in animals [137], and renin is a crucial enzyme whose inhibition is considered as a useful approach to treat hypertension. Few studies have analyzed the inhibitory effects of EGCG on renin activity. However, in a recent *in vitro* study, it has been reported inhibitory properties of EGCG with an inhibitory concentration 50 (IC_50_) value of 44.53 μM. Furthermore, this study revealed that EGCG acted in an uncompetitive manner and suggested that galloyl moiety and ortho-trihydroxy phenyl structures might be favorable for the renin-inhibitory activity of EGCG [138]. The beneficial properties of EGCG have been examined on spontaneously hypertensive rats (SHR), a model of hypertension, insulin resistance and obesity. In a study conducted by Potenza *et al.*, it has been showed a significant decrease of blood pressure equivalent in rats treated with 3 mg/kg/day enalapril (an angiotensin converting enzyme inhibitor) and rats treated with 200 mg/kg/day EGCG compared to SHR control. Additionally, this study confirmed that EGCG stimulated nitric oxide production from endothelium through a PI-3-kinase pathway suggesting that EGCG may be relevant to improve symptoms of metabolic syndrome and particularly, hypertension [139].

## 9. Conclusions and Perspectives

There is traditional and widespread use of dietary flavonoids all around the world. While anecdotal and epidemiological evidence has historically supported the idea of a link between varied diet and good health, experimental evidence supports the idea that dietary flavonoids have potentially beneficial effects on a multitude of health conditions, including metabolic syndrome. As discussed in this review, the beneficial properties of EGCG have been established in both various cell lines and different animal models. Studies in cell lines have also demonstrated that these compounds can affect a range of signaling and metabolic pathways resulting in improving various symptoms including endothelial dysfunction.

In recent years, evidence has suggested that DNA methylation is involved in the emergence of metabolic syndrome through the epigenetic regulation of numerous candidate genes. Thus, a particular attention has been focused on epigenetic modulations induced by obesity. In fact, cell studies showed methylation variations in genes involved in energy metabolism such as *ppar-*α, *ucp1* and *phosphoenolpyruvate carboxinase* [140]. Furthermore, the genes of *leptin receptor* and *leptin* have been found to be mutated in obese individuals [141]. Hypertension, another symptom of metabolic syndrome, showed variations in DNA modulations since it has been reported in hypertensive rat models a hypomethylation of the *(pro)renin* gene [142] or of the *adrenergic* β*1* gene [143]. Interestingly, recent studies have described the beneficial properties of flavonoids to prevent obesity or hypertension through a regulation of DNA methylation patterns [144,145]. Regarding EGCG, the most existing studies have focused on the modulation of DNA methylation in tumorigenesis suggesting interesting scientific opportunities to determine the properties of EGCG in DNA methylation, particularly in metabolic syndrome.

On the basis of these results, one can advance the notion that EGCG is readily available and widely consumed and may have a high potential use in the prevention of metabolic syndrome. Nevertheless, the preventive activity of this compound has not been consistently observed in human studies. Although some clinical studies have evaluated the preventive properties of EGCG in obesity (Table 3), other clinical studies should be considered in order to provide conclusions about the use of EGCG to prevent all the symptoms of the metabolic syndrome.

**Table 3:** Properties of EGCG on human obesity.

Subjects	Dose	Duration	Results	Ref
115 obese women		12 weeks	↓ body weight ↓ BMI ↓ total cholesterol ↓ LDL cholesterol	[146]
56 obese, hypertensive patients	379 mg/day	12 weeks	↓ SBP, ↓ DBP ↓ serum glucose ↓ insulin resistance ↓ LDL cholesterol ↓ TG	[147]
46 obese patients	379 mg/day	12 weeks	↓ BMI ↓ body weight ↓ serum glucose ↓ total cholesterol ↓ LDL cholesterol ↓ TG	[148]
35 obese patients with MS	870 mg/day	8 weeks	↓ body weight ↓ BMI ↓ LDL cholesterol ↓ LDL/HDL ratio	[149]
88 obese patients	800 mg/day	8 weeks	↓ DBP	[150]
40 obese children	576 mg/day	24 weeks	↓ body weight ↓ SBP ↓ LDL cholesterol	[151]

BMI: body mass index; LDL: low density lipoprotein; SBP: systolic blood pressure; DBP: diastolic blood pressure; MS: metabolic syndrome; TG: triglycerides.
